# Lentigo maligna mimicking invasive melanoma in Mohs surgery: a case report

**DOI:** 10.12688/f1000research.3-25.v1

**Published:** 2014-01-24

**Authors:** Teresa Tsakok, Nisith Sheth, Alistair Robson, Catherine Gleeson, Raj Mallipeddi

**Affiliations:** 1 Dermatological Surgery and Laser Unit, St John’s Institute of Dermatology, St Thomas’ Hospital, London, UK

## Abstract

Lentigo maligna is a lentiginous proliferation of atypical melanocytes confined to the epidermis, typically on chronically sun-damaged skin. Following biopsy and exclusion of invasive disease, therapy may involve Mohs surgery, topical treatment or radiotherapy. However, lentigo maligna often involves adnexal structures, creating histological difficulty in distinguishing these foci from invasive melanoma. We present a case in which, during Mohs excision, a nodule of severely atypical melanocytes appeared to lie within the dermis, potentially altering treatment and prognosis. The use of laminin-5 provided a means of resolving this diagnostic dilemma, facilitating continuation of Mohs surgery until tumour clearance was achieved.

## Introduction

Lentigo maligna is a form of melanoma *in-situ* that may be present in up to 1.17% of Caucasians aged 65–74 years ^1^. The most significant risk factor is lifetime UV radiation exposure; accordingly, lentigo maligna often presents as an irregularly pigmented macule at sun-damaged sites in elderly individuals – particularly the head and neck. Left untreated, reported rates of progression to invasive melanoma vary between 2.2–50% ^2^, and the prognosis of invasive disease is similar to that of other melanoma types after adjusting for tumour thickness ^3^. However, controversy remains regarding the best therapeutic modality for lentigo maligna.

## Report

A 76 year old British seaman presented in April 2010 with an unevenly pigmented brown macule on the left side of his neck, measuring 31 × 26 mm. This was at the site of a previous lentigo maligna that had been excised with a 5 mm margin.

**Figure 1.**
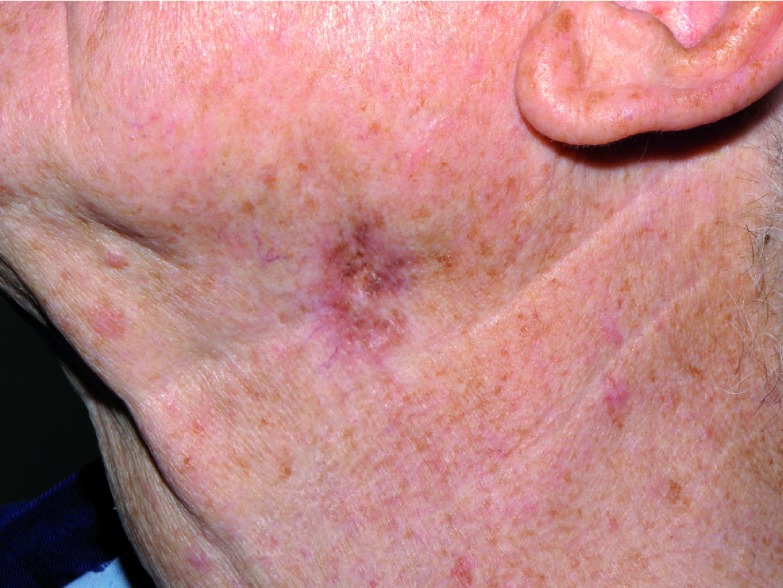
Lesion on left side of neck.

A diagnostic biopsy showed a lentiginous proliferation of atypical melanocytes tracking down adnexae, confirming recurrence. The patient was referred for Mohs surgery.

In addition to lentigo maligna in the epidermis, one Mohs section revealed a large nodule of severely atypical melanocytes, apparently lying mid-dermis.

**Figure 2.**
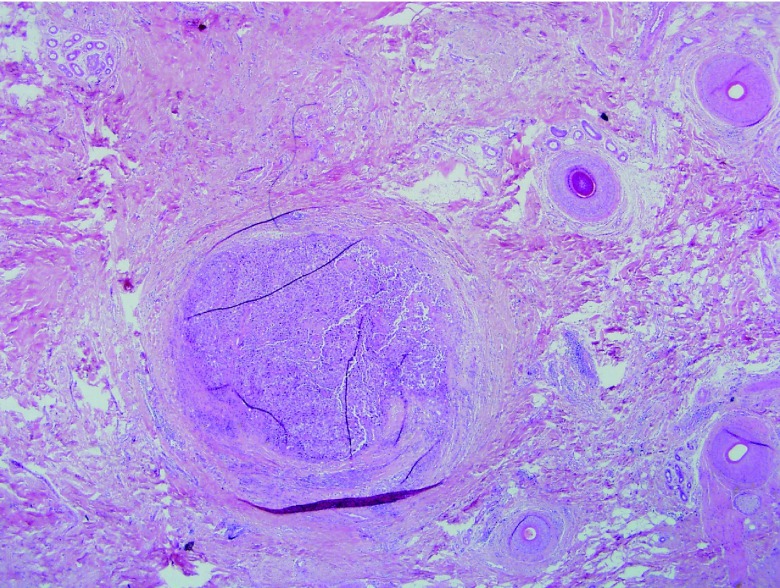
Nodule of atypical melanocytes (H&E ×20).

This presented a significant interpretational problem. Lentigo maligna commonly involves hair follicle epithelia; thus, the nodule may reflect complete replacement of the follicular epithelial cells still confined by basement membrane ( *in-situ* disease). Alternatively, it could represent a focus of invasive melanoma, in which case Mohs surgery would have to be aborted.

To clarify this important distinction, immunocytochemical staining was performed for laminin-5 (Dako, Z0097, 1:100), a component of basement membrane. This revealed immunopositivity of the basement membrane of the follicular epithelium, confirming that the atypical melanocytes were confined to the hair follicle.

**Figure 3.**
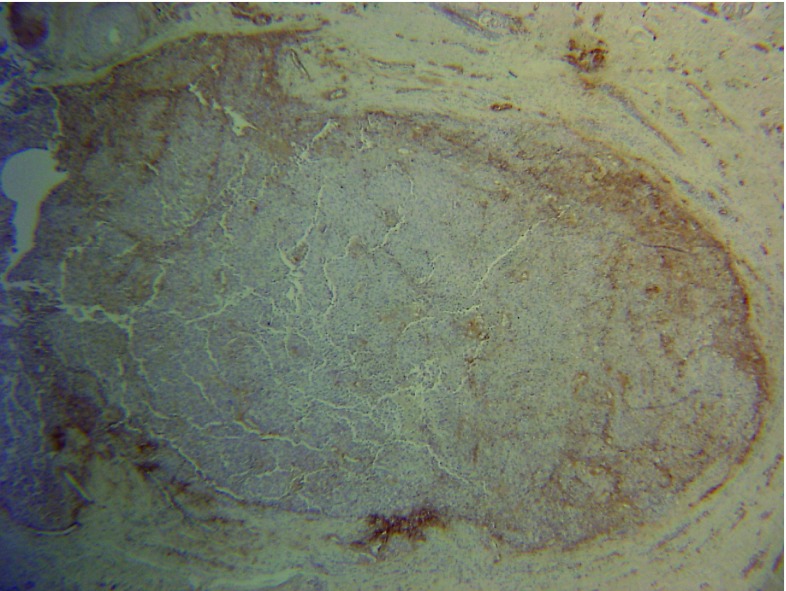
Nodule with laminin 5 staining (×20).

Mohs surgery was therefore completed in a 2-stage, 10-section procedure. Following confirmation of negative margins, the defect measured 45 × 35 mm – substantially larger than the original lesion. This was closed using a rhombic transposition flap, which healed well. To date there has been no recurrence.

## Discussion

Lentigo maligna presents several challenges in terms of diagnosis and management. Firstly, clinical and dermoscopic features may be strikingly similar to solar lentigo ^4^. It is also prone to significant subclinical extension beyond the visible limits of the tumour. Following initial tumour debulk, single atypical melanocytes scattered irregularly along the dermo-epidermal junction may be the only histological feature of residual lentigo maligna ^5^. This difficulty is compounded by the presence of single mildly atypical melanocytes in chronically sun-damaged skin ^6^. Finally, lentigo maligna often spreads into deep adnexal structures, confounding both diagnosis and treatment ^6^.

There are various management options for lentigo maligna. Excision is accepted as the gold standard and can consist of conventional excision with a 5 mm margin, or some form of Mohs surgery. Other therapies are destructive, including radiotherapy and imiquimod (Aldara), a topical immunomodulator.

An evidence-based comparison of treatment modalities supports Mohs surgery as first-line, with recurrence rates as low as 0.3% compared to up to 20% for standard excision ^6^. The follow-up periods of these trials varied from 18 months to almost five years, but it is notable that the most favourable study for Mohs surgery included 625 patients and measured recurrence over 58 months ^7^.

The impressive statistics for Mohs surgery are in part attributable to the superior histological margin control afforded by this technique. Traditional surgical excision uses ‘bread-loafing’ to check for residual tumour, but this allows visualization of less than 1% of the margin. By contrast, in Mohs surgery, tissue is excised tangentially before being processed into horizontal sections. This method – combined with precise mapping and orientation of tissue by the Mohs surgeon reading the pathology – allows all tumour extensions to be traced. The end benefits are two-fold: recurrence rates are significantly improved, as tumour clearance is optimised; and healthy tissue is conserved, preserving cosmesis and function – important considerations on the face. The significance of visualising the entire tumour is underscored by evidence showing that almost 25% of biopsy-proven melanoma *in-situ* lesions are found to contain invasive melanoma upon pathological examination after complete surgical removal ^6^.

Follicular involvement by lentigo maligna is a widely recognised phenomenon, and is a characteristic of lentiginous spread. Indeed, a key dermoscopic feature of lentigo maligna is asymmetrical pigmentation of follicular openings, reflecting uneven descent of melanoma cells into individual hair follicles. Over time the slate-grey dots and streaks surrounding the follicle progress into a rhomboidal pattern, and then to eventual obliteration of the follicle, visible on dermoscopy as black blotches highly specific for malignant growth.

Follicular melanoma has been reported as a rare variant of melanoma, to be distinguished from lentigo maligna melanoma by its significantly smaller surface area (typically < 0.5 cm), the relative symmetry of the lesion and its similarity in appearance to a comedo or pigmented cyst ^8^. A recent study investigating the growth characteristics of melanoma *in-situ* in relation to hair follicle microanatomy has suggested that the follicle itself represents a physiological barrier, restricting the intraepithelial spread of melanoma tumour cells at or beyond the level of the hair follicle bulge ^9^.

The present case posed an interesting diagnostic dilemma – the difficulty in distinguishing between dermal invasion and melanoma *in-situ* within a hair follicle. To resolve this, we used immunocytochemistry for laminin-5 to delineate normal basement membrane surrounding the hair follicle, indicating *in-situ* disease and therefore justifying continuation of the Mohs procedure.

Laminin-5 is a component of the adhesion complex in basement membrane ^10^. It is thought to act as a ligand for the attachment of normal melanocytes to the basement membrane. Conversely, loss of laminin production by melanoma cells is thought to be a marker for malignant transformation ^10^. To our knowledge, the use of laminin-5 staining during Mohs has not been previously reported.

## Learning points

- Lentigo maligna presents significant challenges in diagnosis and treatment
- Mohs surgery can be effective as long as disease remains *in-situ*, but this may be difficult to ascertain
- Almost 25% of biopsy-proven melanoma *in-situ* lesions are upstaged to invasive melanoma upon pathological investigation, highlighting the importance of visualising the entire tumour
- Surgeons using the Mohs technique require a thorough understanding of histopathology using horizontal sections to achieve meaningful margin control

## Consent

Written informed consent for publication of clinical details and clinical images was obtained from the patient.

## References

[1] Weinstock MA Sober AJ: The risk of progression of lentigo maligna to lentigo maligna melanoma. *Br J Dermatol.* 1987;16:303–10 3567069 10.1111/j.1365-2133.1987.tb05843.x

[2] Wayte DM Helwig EB: Melanotic freckle of Hutchinson. *Cancer.* 1968;21:893–911 5649048 10.1002/1097-0142(196805)21:5<893::aid-cncr2820210513>3.0.co;2-8

[3] Smalberger GJ Siegel DM Khachemoune A: Lentigo maligna. *Dermatol Ther.* 2008;21(6):439–46 10.1111/j.1529-8019.2008.00244.x 19076621

[4] Cohen LM: Lentigo maligna and lentigo maligna melanoma. *J Am Acad Dermatol.* 1995;33(6):923–936quiz 937–40. 10.1016/0190-9622(95)90282-1 7490362

[5] Barlow RJ White CR Swanson NA: Mohs' micrographic surgery using frozen sections alone may be unsuitable for detecting single atypical melanocytes at the margins of melanoma *in-situ*. *Br J Dermatol.* 2002;146(2):290–4 10.1046/j.1365-2133.2002.04661.x 11903242

[6] Dawn ME Dawn AG Miller SJ: Mohs surgery for the treatment of melanoma *in-situ*: a review. *Dermatol Surg.* 2007;33(4):395–402 10.1111/j.1524-4725.2007.33085.x 17430372

[7] Bricca GM Brodland DG Ren D: Cutaneous head and neck melanoma treated with Mohs micrographic surgery. *J Am Acad Dermatol.* 2005;52:92–100 10.1016/j.jaad.2004.08.038 15627086

[8] Hantschke M Mentzel T Kutzner H: Follicular malignant melanoma: a variant of melanoma to be distinguished from lentigo maligna melanoma. *Am J Dermatopathol.* 2004;26(5):359–63 15365365 10.1097/00000372-200410000-00002

[9] Pozdnyakova O Grossman J Barbagallo B: The hair follicle barrier to involvement by malignant melanoma. *Cancer.* 2009;115(6):1267–75 10.1002/cncr.24117 19152437

[10] Scott GA Cassidy L Tran H: Melanocytes adhere to and synthesize laminin-5 *in vitro*. *Exp Dermatol.* 1999;8(3):212–21 10389639 10.1111/j.1600-0625.1999.tb00373.x

