# Supplementary material for: Lentigo maligna mimicking invasive melanoma in Mohs surgery: a case report
Source: F1000Res. 2014 Jan 24;3:25. [Version 1] doi: 10.12688/f1000research.3-25.v1 (PMC4097356; doi:10.12688/f1000research.3-25.v1)
Supplement: Peer review history

# Peer review: Referee response for version 1

**DOI:** 10.5256/f1000research.3409.r4667

**Published:** 2014-05-30

**Authors:** Rajiv Kumar

**Affiliations:** 1 Germany Cancer Research Center, Helmholtz Association, Heidelberg, Germany

Well defined report. I have no further comments.

I have read this submission. I believe that I have an appropriate level of expertise to confirm that it is of an acceptable scientific standard.

# Peer review: Referee response for version 1

**DOI:** 10.5256/f1000research.3409.r3331

**Published:** 2014-02-10

**Authors:** Eduardo Nagore

**Affiliations:** 1 Department of Oncology, Valencian Institute of Oncology, Valencia, Spain

A nice case report - I have no comments.

I have read this submission. I believe that I have an appropriate level of expertise to confirm that it is of an acceptable scientific standard.
